# Construction strategies and implementation pathways of resilience governance system for care risks in older adult care institutions from a social-ecological perspective

**DOI:** 10.3389/fpubh.2026.1853068

**Published:** 2026-08-20

**Authors:** Chunyan Li, Yang Xie, Weiping Liu

**Affiliations:** 1 School of Nursing, Hunan University of Chinese Medicine, Changsha, Hunan, China; 2 Xiangnan University, Chenzhou, Hunan, China

**Keywords:** care risks, complex adaptive systems, older adult care institutions, resilience governance, social-ecological systems

## Abstract

Against the backdrop of population ageing, the governance of care risks in older adult care institutions has become a global challenge. This paper aims to construct a resilience governance system for care risks in older adult care institutions based on social-ecological systems theory, exploring its construction strategies and implementation pathways. The article first reviews the development trajectory of resilience governance theory in social sciences, then introduces social-ecological systems theory into the research on risk governance in older adult care institutions, constructing a resilience governance framework encompassing macro, meso, and micro levels. The study proposes a three-dimensional construction strategy of ‘concept-element-mechanism’, including three core concepts and three operational mechanisms. Regarding implementation pathways, the article demonstrates a trinity implementation strategy of strengthening resilience governance subject coordination, improving resilience governance policy support, and digital technology empowerment of resilience governance. International case analyses of older adult care institutions’ responses to the COVID-19 pandemic are presented as illustrative examples demonstrating the framework’s relevance and applicability, rather than as formal empirical validation. Research indicates that the resilience governance system from a social-ecological perspective can effectively enhance the capacity of older adult care institutions to respond to care risks, providing theoretical basis and practical guidance for constructing a sustainable older adult care service system. The innovation of this paper lies in combining social-ecological systems theory with resilience theory, constructing a systematic, dynamic, and adaptive governance framework, opening up new theoretical perspectives for research on risk governance in older adult care institutions exploring its construction strategies and implementation pathways. The article first reviews the development trajectory of resilience governance theory in social sciences, then introduces social-ecological systems theory into the research on risk governance in older adult care institutions, constructing a resilience governance framework encompassing macro, meso, and micro levels. The study proposes a three - dimensional construction strategy of “concept-element-mechanism,” including three core concepts: development-security co-construction, temporal–spatial integration, and action-governance alignment. These concepts should emerge from co-creation with multiple stakeholders (e.g., older adult residents, caregivers, families) to ensure they reflect diverse values. Beyond risk mitigation, the framework prioritizes wellbeing, dignity, and a sense of meaning—key values for human-centered resilience in older adult care. Additionally, as well as three operational mechanisms: adaptive proactive circulation mechanism, full-element resilience coupling mechanism, and whole-process dynamic response mechanism. Regarding implementation pathways, the article demonstrates a trinity implementation strategy of strengthening resilience governance subject coordination, improving resilience governance policy support, and digital technology empowerment of resilience governance. Through case analysis of global older adult care institutions’ responses to the COVID-19 pandemic, this paper validates the effectiveness of the resilience governance system and proposes localization recommendations combined with Chinese practice. Research indicates that the resilience governance system from a social-ecological perspective can effectively enhance the capacity of older adult care institutions to respond to care risks, while acknowledging the necessary trade-offs between resilience and other priorities (e.g., cost, equity) in resource-constrained settings, providing theoretical basis and practical guidance for constructing a sustainable older adult care service system. The innovation of this paper lies in combining social- ecological systems theory with resilience theory, constructing a systematic, dynamic, and adaptive governance framework, opening up new theoretical perspectives for research on risk governance in older adult care institutions.

## 1 Introduction

The global population ageing process continues to accelerate, becoming one of the most significant social transformations of the 21st century. According to the United Nations’ “World Population Prospects 2022” (1) report, the proportion of the global population aged 65 and above is projected to increase from 10% in 2022 to 16% by 2050, when the population aged 65 will reach 1.6 billion. In China, population ageing exhibits characteristics of rapid pace, large scale, and ageing before affluence. Data from the Seventh National Population Census (2) shows that China’s population aged 60 and above has reached 264 million, accounting for 18.7% of the total population, with those aged 65 and above numbering 191 million, accounting for 13.5%. It is projected that by 2035, China’s population aged 60 and above will exceed 400 million, accounting for more than 30%, entering a deeply aged society (3).

Against the backdrop of population ageing, older adult care institutions, as an important component of the older adult care service system, undertake the important function of providing professional care services for disabled, semi-disabled, and older adults of advanced age. However, older adult care institutions face multiple care risks during operation, including safety risks such as falls, pressure ulcers, aspiration, and wandering of older adults, risks of infectious disease transmission, and mental health risks (4). These risks not only threaten the life and health of older adults but also bring enormous challenges to the operational management of older adult care institutions. The outbreak of the COVID-19 pandemic has further highlighted the vulnerability of older adult care institutions in responding to public health emergencies. Research shows that in 2020, COVID-19 deaths in older adult care institutions in European and American countries accounted for 30–60% of total deaths, making older adult care institutions a key focus and difficulty in pandemic prevention and control (5).

Traditional risk governance models for older adult care institutions are primarily based on risk prevention and control approaches, emphasizing reducing the probability of risk occurrence through formulating rules and regulations, strengthening supervision and inspection, and improving the professional skills of nursing staff. However, this static, unidirectional governance model often proves inadequate when facing complex and variable care risks. In recent years, the concept of resilience has gradually been introduced into the field of social governance, providing a new theoretical perspective for responding to uncertainty and complexity. Resilience originally stems from the fields of ecology and engineering, referring to a system’s ability to recover to its original state or adapt to new environments after experiencing disturbances (6). In the field of social governance, resilience is understood as the absorption capacity, adaptive capacity, and transformation capacity of social systems when facing shocks and pressures (7).

Social-ecological systems theory provides a systematic analytical framework for understanding care risks in older adult care institutions. Crucially, this framework must incorporate temporal dynamics, distinguishing between fast variables (e.g., rapid spread of infections) and slow variables (e.g., institutional adaptation, policy adjustments). Resilience governance must account for both immediate shocks and gradual systemic adaptations. Furthermore, managing deep uncertainty—a defining feature of complex systems—requires adaptive management principles, emphasizing flexibility, learning-by-doing, and iterative policy adjustments under uncertainty (8).

This theory emphasizes the interdependence and co-evolution of human social systems and natural ecological systems, viewing social systems as complex adaptive systems with characteristics such as multi-level structure, dynamism, and self-organization (9). Applying social-ecological systems theory to research on risk governance in older adult care institutions helps us understand the generation mechanism and evolution patterns of care risks from a holistic, relational, and dynamic perspective, constructing a more adaptive and resilient governance system.

This paper aims to answer the following core questions: From the perspective of social-ecological systems theory, how can we construct a resilience governance system for care risks in older adult care institutions? What are its core elements, operational mechanisms, and implementation pathways? To answer these questions, this paper first reviews the development trajectory of resilience governance theory in social sciences, exploring its epistemological and ontological foundations; second, introduces social-ecological systems theory into research on risk governance in older adult care institutions, constructing a multi-level resilience governance framework; third, proposes construction strategies and operational mechanisms for the resilience governance system; finally, based on international experience and Chinese practice, explores implementation pathways for the resilience governance system. The innovation of this paper lies in combining social-ecological systems theory with resilience theory, providing new theoretical perspectives and analytical tools for research on risk governance in older adult care institutions, and constructing a systematic and operational governance framework.

## 2 Methodology

This study employs a narrative policy review approach to synthesize theoretical and empirical literature and develop a conceptual framework. Literature was identified through systematic searches of PubMed, Web of Science, and Google Scholar using keywords related to resilience governance, social-ecological systems, older adult care, and COVID-19 pandemic responses. The multi-level social-ecological systems theory provided the organizing framework for synthesizing literature and developing the concept-element-mechanism model. The figures presented in this paper were developed iteratively through literature synthesis and theoretical modeling to represent the logical relationships among governance dimensions. The international case analyses presented in Section 6 serve as illustrative examples rather than empirical validation, selected based on international diversity, COVID-19 response comprehensiveness, and data availability.

## 3 Resilience governance: theoretical foundations and epistemological considerations

Resilience governance, as an emerging governance paradigm, has theoretical foundations traceable to multiple disciplinary fields. In the field of ecology, Holling first proposed the concept of ecological resilience in 1973, defining it as the ability of an ecological system to recover to its original state after experiencing disturbances (6). This concept emphasizes system stability and recovery capacity, referred to as “engineering resilience.” As research deepened, scholars gradually recognized that resilience is not merely about recovering to the original state, but more importantly about a system’s adaptive and transformative capacity when facing disturbances. Walker et al. proposed the concept of “ecological resilience,” emphasizing a system’s ability to transition between multiple stable states and to adapt to change while maintaining basic functions and structures (10).

In the social sciences, the introduction of the resilience concept began in the late 20th and early 21st centuries. Adger defined resilience as “the capacity of social-ecological systems to absorb disturbances and reorganize while maintaining essentially the same function, structure, identity, and feedbacks” (11). This definition emphasizes the interdependence of social and ecological systems, laying the foundation for the development of social-ecological systems theory. In the fields of public management and policy research, resilience is viewed as an important capacity for governments and social organizations to respond to crises and uncertainty. Comfort et al. proposed the concept of “adaptive resilience,” emphasizing organizational learning and innovation capacity when facing dynamically changing environments (12).

The development of resilience governance theory has undergone an evolution from “bounce-back resilience” to “adaptive resilience” and then to “transformative resilience.” Bounce-back resilience emphasizes a system’s ability to recover to its original state after experiencing shocks, assuming the existence of an ideal stable state to which the system aims to return as quickly as possible. However, in the complex and changing modern society, this static view of resilience can no longer meet governance needs. Adaptive resilience emphasizes a system’s adjustment and adaptation capacity when facing continuous change; the system does not necessarily need to recover to its original state but adapts to new environments by adjusting structure and function. Transformative resilience goes further, emphasizing a system’s transformation capacity when facing fundamental changes, achieving fundamental system transformation through innovation and restructuring (13).

**Figure 1:**
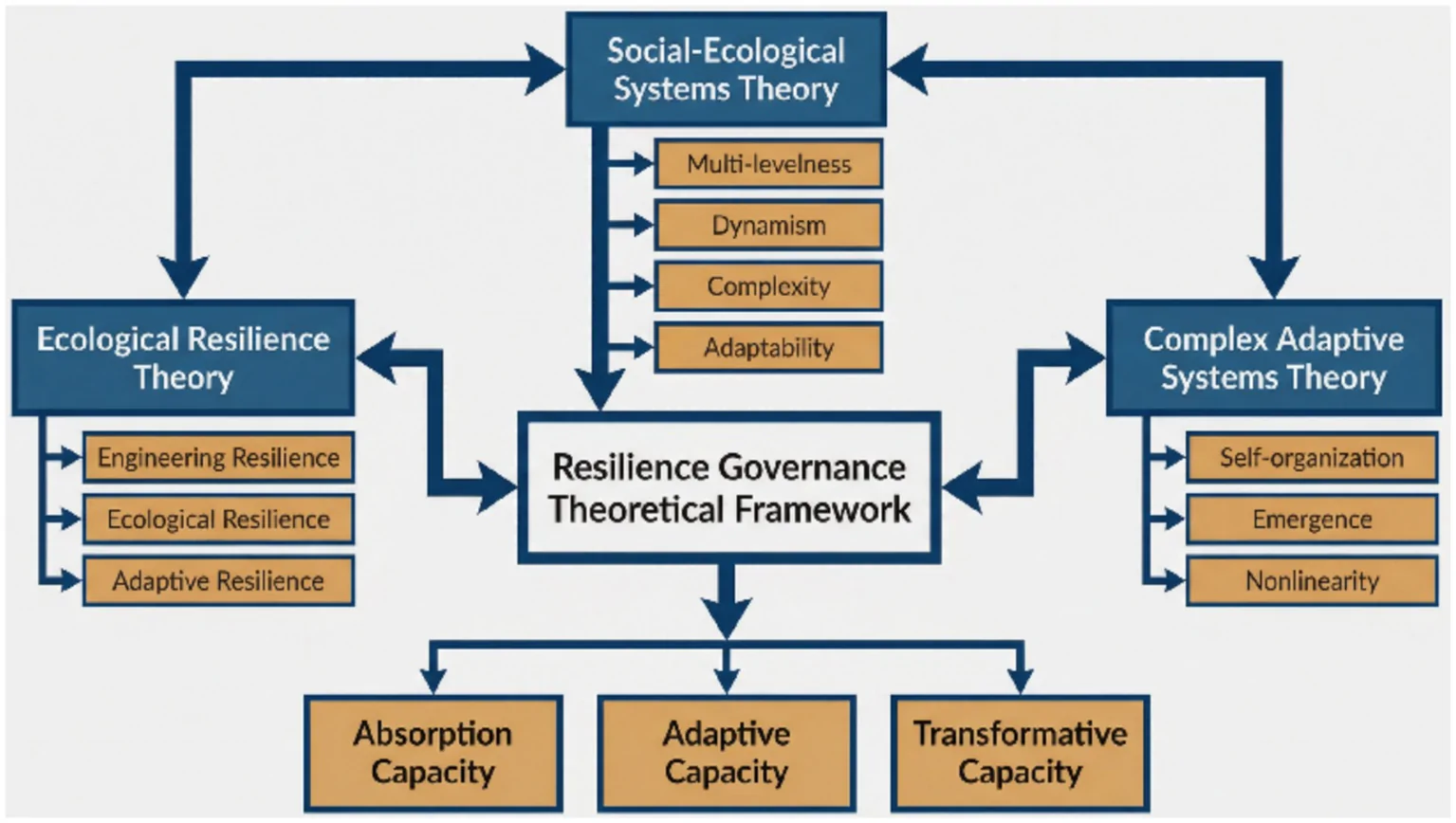
Theoretical framework of resilience governance.

Figure 1 presents the theoretical foundations of resilience governance, including three theoretical pillars: ecological resilience theory, social-ecological systems theory, and complex adaptive systems theory, as well as the resulting three system capacities: absorption capacity, adaptive capacity, and transformation capacity.

At the epistemological level, resilience governance theory embodies a shift from simplicity thinking to complexity thinking. Traditional risk governance models are based on linear causal relationships and predictability assumptions, believing that risks can be effectively controlled by identifying risk factors and formulating response measures. However, social systems in reality are complex adaptive systems with characteristics such as non-linearity, emergence, and self-organization, making system behavior difficult to predict and control through simple causal relationships (14). Resilience governance theory acknowledges system complexity and uncertainty, emphasizing responding to unpredictable risks and challenges by enhancing system adaptive capacity and transformation capacity.

At the ontological level, resilience governance theory’s understanding of social reality embodies characteristics of social constructivism. As Berger and Luckmann elaborated in “The Social Construction of Reality,” social reality is formed through human interaction and meaning construction (15). Resilience governance theory holds that system resilience depends not only on objective material conditions and technical capabilities but more importantly on social actors’ perceptions of risk, understanding of resilience, and governance culture and institutional arrangements formed in practice. This view aligns with social-ecological systems theory, which emphasizes the mutual construction and co-evolution of human social systems and natural ecological systems.

When applying resilience governance theory to care risk governance in older adult care institutions, the particularity of older adult care institutions as social-ecological systems must be considered. Older adult care institutions are complex systems with multi-level, multi-actor, and multi-element interactions, including multiple stakeholders such as older adults, nursing staff, management personnel, family members, government regulatory departments, and communities. Complex power relationships, resource allocation mechanisms, and cultural norms exist within the system. Meanwhile, older adult care institutions are also influenced by external environments, including policies and regulations, economic conditions, technological development, and social culture. Therefore, constructing a resilience governance system for care risks in older adult care institutions requires starting from a holistic social-ecological systems perspective, considering interactions among internal system elements and the interactive relationship between the system and external environment.

## 4 Social-ecological systems theory and care risk governance in older adult care institutions

Social-ecological systems theory provides an integrative analytical framework for understanding care risks in older adult care institutions. This theory originated from ecologists’ research on the interaction between human activities and natural ecological systems and has been widely applied to fields such as resource management, environmental governance, and community development (16). In recent years, social-ecological systems theory has gradually been introduced into ageing research and older adult care service research, providing new perspectives for understanding the health and wellbeing of older adults (17).

The core viewpoint of social-ecological systems theory is that human social systems and natural ecological systems are interdependent, co-evolving complex systems. Systems consist of multiple levels, including micro-level (individuals), meso-level (organizations and communities), and macro-level (institutions and policies), with complex interactions and feedback mechanisms among levels (18). Systems have several important characteristics:

First, *multi-level structure*. Social-ecological systems consist of multiple nested levels, each with its specific structure, function, and dynamic characteristics. In care risk governance in older adult care institutions, the micro-level includes the health status of older adult care, professional capabilities of nursing staff, and specific care practices; the meso-level includes organizational structure, management systems, resource allocation, and community environment of older adult care institutions; the macro-level includes national older adult care policies, laws and regulations, economic development level, and social culture. Levels interact with each other, jointly determining the system’s resilience level.

Second, *dynamism*. Social-ecological systems are in constant change, with system states, structures, and functions evolving over time. This dynamism comes both from internal self-organization processes and external environmental disturbances. In older adult care institutions, the health status of older adults changes with age, nursing staff turnover occurs, management systems adjust, and policies and regulations update. Resilience governance needs to adapt to this dynamism, establishing mechanisms for continuous learning and adjustment.

Third, *complexity*. Social-ecological systems consist of numerous elements with non-linear interactions among them, making system behavior difficult to predict through simple causal relationships. In older adult care institutions, the generation of care risks is often the result of multiple factors acting together, including the physiological condition, psychological state, social support, physical environment, care quality, and management level of older adults. Complex interactions exist among these factors; a change in one factor may trigger a chain reaction, leading to sudden changes in system state.

Finally, *adaptability*. Social-ecological systems have self-regulation and adaptation capacity to change. When systems experience disturbances, they can adapt to new environmental conditions by adjusting internal structure and function. This adaptive capacity is an important source of system resilience. In older adult care institutions, adaptability manifests in multiple aspects, such as nursing staff adjusting care plans according to the needs of older adults, management personnel optimizing resource allocation according to operational conditions, and policymakers adjusting older adult care policies according to social changes.

Based on social-ecological systems theory, we can divide the care risk governance system of older adult care institutions into three levels: macro-level, meso-level, and micro-level.

**Figure 2:**
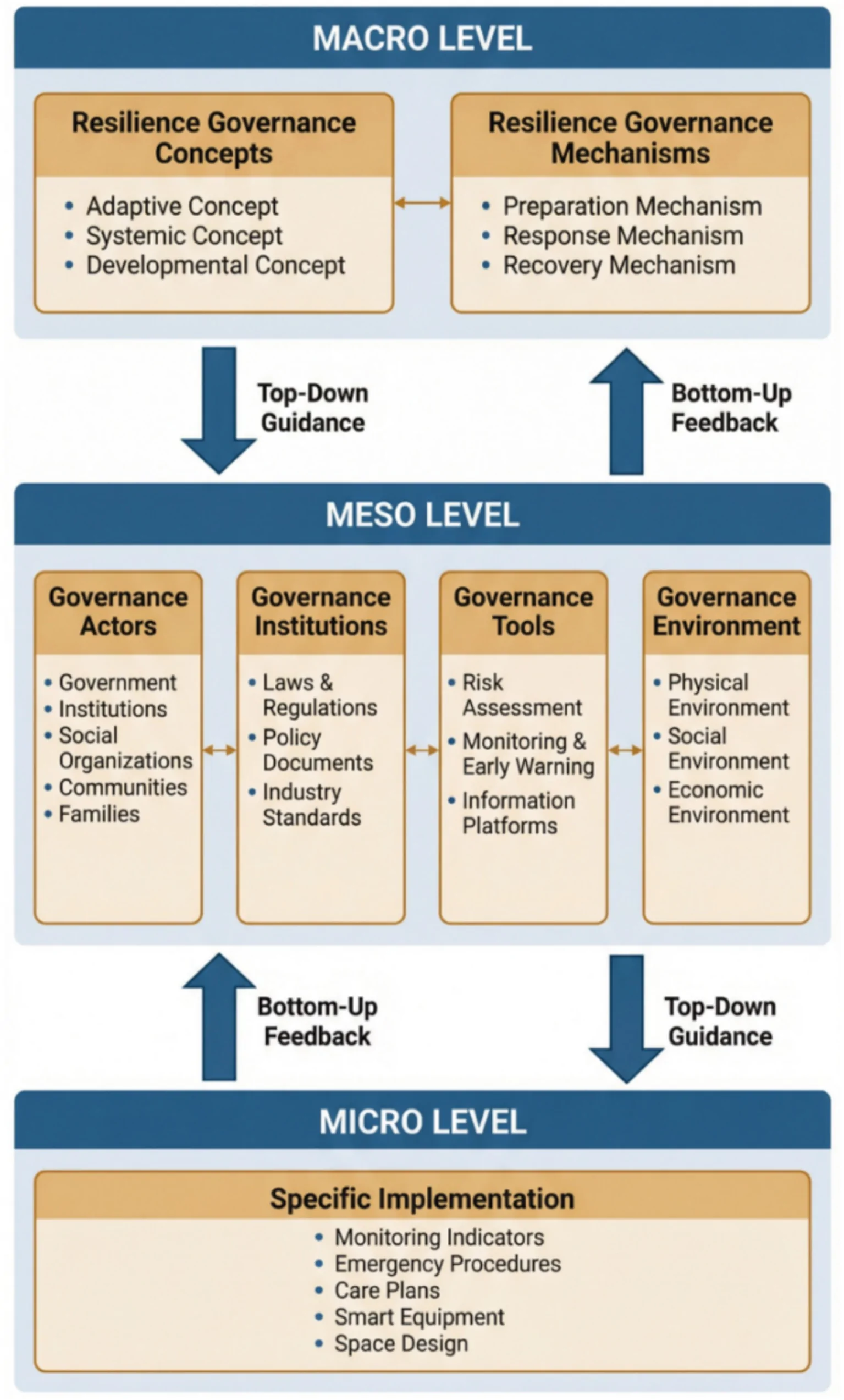
Multi-level resilience governance system architecture for older adult care institutions.

Figure 2 presents the multi-level architecture of the resilience governance system for older adult care institutions. The macro-level includes resilience governance concepts and mechanisms; the meso-level includes four major elements: governance subjects, institutions, tools, and environment; the micro-level covers specific implementation content. The three levels form dynamic interactions through downward guidance and upward feedback.

The macro-level includes resilience governance concepts and resilience governance mechanisms. Resilience governance concepts refer to values and principles guiding the entire governance system, including adaptive concepts, systemic concepts, and developmental concepts. The adaptive concept emphasizes the system’s capacity to respond to uncertainty and change, requiring governance subjects to have flexible thinking and the ability to adjust strategies promptly according to environmental changes. The systemic concept emphasizes understanding care risks from a holistic and relational perspective, recognizing that risk generation and evolution result from the interaction of multiple factors. The developmental concept emphasizes achieving continuous improvement and innovation of the system in the process of responding to risks, rather than merely recovering to the original state. Resilience governance mechanisms include preparation mechanisms, response mechanisms, and recovery mechanisms, corresponding to the three stages of risk governance: before, during, and after events.

The meso-level includes resilience governance subjects, resilience governance institutions, resilience governance tools, and resilience governance environment. Resilience governance subjects refer to various actors participating in care risk governance, including government departments, older adult care institutions, social organizations, communities, families, and older adults themselves. The collaborative participation of multiple subjects is an important characteristic of resilience governance. Resilience governance institutions refer to formal and informal rules regulating governance behavior, including laws and regulations, policy documents, industry standards, organizational charters, and cultural norms. Resilience governance tools refer to technical means and methods supporting governance activities, including risk assessment tools, monitoring and early warning systems, information management platforms, and intelligent care equipment. Resilience governance environment refers to external conditions affecting governance effectiveness, including physical environment (such as architectural design and facilities of older adult care institutions), social environment (such as community support and social capital), and economic environment (such as funding input and market mechanisms).

The micro-level is the implementation of macro concepts and meso elements in specific practice, including specific risk monitoring indicators, emergency operation procedures, personalized care plans, intelligent equipment applications, and physical space design. The implementation quality at the micro-level directly determines the effectiveness of the resilience governance system.

Complex interactive relationships exist among these three levels. Macro-level concepts and mechanisms provide guidance for meso-level element configuration; meso-level subjects, institutions, tools, and environment provide support for micro-level practice; micro-level practice effects feed back to meso and macro levels, promoting continuous system improvement. This multi-level interaction embodies the complexity and dynamism of social-ecological systems.

### 4.1 Construction strategies of resilience governance system: concepts, elements, and mechanisms

Based on social-ecological systems theory and resilience governance theory, this paper proposes a three-dimensional construction strategy of “concept-element-mechanism” for the resilience governance system of care risks in older adult care institutions. This strategic framework embodies logical levels from abstract to concrete, from concept to practice, providing guidance for constructing a systematic and operational resilience governance system.

#### 4.1.1 Construction concepts: value orientation of resilience governance

The construction of a resilience governance system first requires establishing core concepts, which are value orientations guiding the design and operation of the entire governance system. This paper proposes three core concepts: development-security co-construction, temporal–spatial integration, and action-governance alignment.

The *development-security co-construction concept* emphasizes simultaneously considering security guarantee and risk prevention and control in the development process of older adult care institutions. Traditional risk governance often views development and security as opposing relationships, believing that strengthening security management constrains development efficiency. However, resilience governance theory holds that development and security are mutually reinforcing; security is the premise of development, and development provides resource guarantee for security. In older adult care institutions, this concept requires embedding flexible adaptation concepts into the care risk governance system, simultaneously considering risk prevention and control needs in institutional development planning, and incorporating security resilience indicators into service quality evaluation systems. For example, in the architectural design of older adult care institutions, not only functionality and comfort should be considered, but also security needs such as emergency evacuation and isolation treatment; in personnel allocation, not only daily care needs should be met, but also redundant capacity for emergency response should be reserved.

The *temporal–spatial integration concept* emphasizes constructing a resilience governance system from full-time-sequence and full-space dimensions. Full-time-sequence refers to a whole-process governance mechanism covering pre-event prevention, during-event response, and post-event recovery. Pre-event prevention includes risk identification, monitoring and early warning, training and drills; during-event response includes emergency activation, coordinated response, and resource allocation; post-event recovery includes facility repair, psychological counseling, and summary improvement. Full-space refers to a governance architecture covering micro, meso, and macro levels. The micro-level focuses on individual older adults’ care needs and risk status; the meso-level focuses on organizational management and community environment of older adult care institutions; the macro-level focuses on policy systems and social support. The temporal–spatial integration concept requires connecting different time-sequence stages and different spatial levels, establishing functional diversity and connectivity, forming a three-dimensional governance network.

The *action-governance alignment concept* emphasizes clarifying and coordinating the demands and responsibilities of different subjects, constructing an action framework that can mobilize and coordinate multiple forces. Care risk governance in older adult care institutions involves multiple stakeholders, including government regulatory departments, older adult care institution managers, nursing staff, older adults and their families, and community organizations. Different subjects have different goals and interest demands: governments focus on public safety and social stability; institutions focus on operational efficiency and economic benefits; nursing staff focus on workload and career development; older adults and families focus on care quality and quality of life. The action-governance alignment concept requires establishing consultation mechanisms for stakeholders, clarifying the boundaries of rights and responsibilities of governance subjects, and promoting multi-party coordination through institutional arrangements and incentive mechanisms, forming governance synergy.

### 4.2 Construction elements: systematic support for resilience governance

The construction of a resilience governance system requires integrating multiple elements to form a systematic support system. Based on the multi-level framework of social-ecological systems theory, this paper elaborates on construction elements of resilience governance from macro, meso, and micro levels.

At the *macro-level*, resilience governance concepts and resilience governance mechanisms are core elements. Resilience governance concepts include adaptive concepts, systemic concepts, and developmental concepts. The adaptive concept requires governance subjects to have flexible thinking and the ability to adjust strategies promptly according to environmental changes, rather than rigidly executing established rules. The systemic concept requires understanding care risks from a holistic and relational perspective, recognizing that risk generation and evolution result from the interaction of multiple factors, requiring systematic response measures. The developmental concept requires achieving continuous improvement and innovation of the system in the process of responding to risks, enhancing system resilience level through learning and reflection. Resilience governance mechanisms include preparation mechanisms, response mechanisms, and recovery mechanisms. Preparation mechanisms include activities such as risk monitoring, early warning, training, and drills, aimed at enhancing system prevention capacity. Response mechanisms include activities such as emergency activation, coordinated response, and resource allocation, aimed at enhancing system response capacity. Recovery mechanisms include activities such as facility repair, psychological counseling, and summary improvement, aimed at enhancing system recovery and learning capacity.

At the *meso-level*, resilience governance subjects, resilience governance institutions, resilience governance tools, and resilience governance environment are key elements. Resilience governance subjects include multiple actors such as government, enterprises, social organizations, grassroots communities, and residents. Government plays a leading role in resilience governance, responsible for policy formulation, resource allocation, and supervision and management. Enterprises (older adult care institutions) are implementation subjects of resilience governance, responsible for specific risk management and care services. Social organizations can provide professional support and social mobilization; grassroots communities can provide neighborhood mutual assistance and social support; residents (older adults and their families) are participants and beneficiaries of resilience governance. The collaborative participation of multiple subjects is an important characteristic of resilience governance. Resilience governance institutions include emergency laws, emergency plans, and emergency culture. Emergency laws provide legal basis and institutional guarantee for resilience governance; emergency plans provide guidance for specific operations; emergency culture provides value support and behavioral norms for resilience governance. Resilience governance tools include technical means such as big data assessment, cloud computing prediction, and artificial intelligence prevention and control. These tools can enhance the accuracy of risk identification, timeliness of early warning, and effectiveness of response. Resilience governance environment includes physical resilience, intelligent monitoring, and policy resource integration. Physical resilience refers to the architectural design and facilities of older adult care institutions being able to support emergency response and risk prevention and control. Intelligent monitoring refers to real-time monitoring of older adults’ health status and environmental safety through sensors and monitoring equipment. Policy resource integration refers to providing sufficient resource guarantee for resilience governance through policy coordination and resource coordination.

At the *micro-level*, specific implementation content is key to the implementation of the resilience governance system. The micro-level includes specific risk monitoring indicators, detailed emergency operation procedures, personalized nursing service plans, specific applications of intelligent equipment, and detailed physical space design. For example, risk monitoring indicators can include vital sign parameters of older adults, activity ability assessment, cognitive function assessment, and mental health assessment; emergency operation procedures can include reporting procedures after risk discovery, activation conditions for emergency response, and disposal steps for different types of risks; personalized nursing plans can formulate differentiated care plans according to the health status, living habits, and cultural background of older adults; intelligent equipment applications can include fall monitoring equipment, vital sign monitoring equipment, intelligent mattresses, and emergency call systems; physical space design can include anti-slip flooring, handrail installation, lighting design, and isolation space design.

### 4.3 Construction mechanisms: dynamic system of resilience governance

The effective operation of a resilience governance system requires establishing scientific operational mechanisms. This paper proposes three core mechanisms: adaptive proactive circulation mechanism, full-element resilience coupling mechanism, and whole-process dynamic response mechanism.

**Figure 3:**
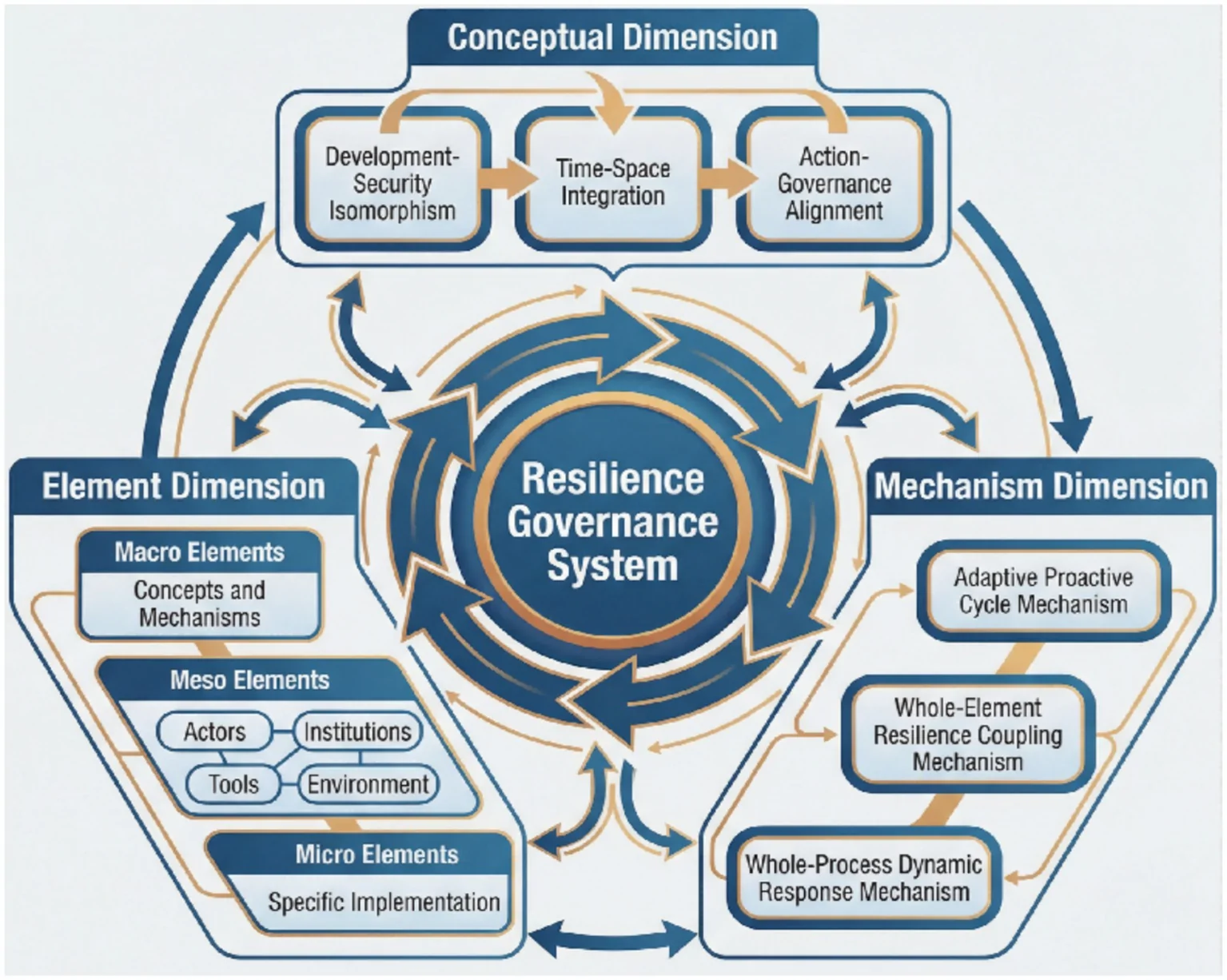
Three-dimensional construction strategy of resilience governance system.

Figure 3 presents the three-dimensional construction strategy of the resilience governance system. The concept dimension includes three core concepts: development-security co-construction, temporal–spatial integration, and action-governance alignment. These concepts are operationalized through specific mechanisms with measurable dimensions: (1) Adaptive proactive circulation mechanism (measured by monitoring frequency, response time to alerts); (2) Full-element resilience coupling mechanism (measured by stakeholder participation rates, resource coordination effectiveness); and (3) Whole-process dynamic response mechanism (measured by response protocol updates, staff training frequency).

The *adaptive proactive circulation mechanism* is a proactive circulation response mechanism established based on learning and innovation feedback capacity. This mechanism includes two links: precise risk identification and deep learning, and innovation incentive and continuous improvement. Precise risk identification and deep learning require organizing cross-professional risk monitoring teams, regularly collecting typical cases, organizing discussion and analysis, creating online and offline learning platforms, promoting knowledge sharing and capacity building. Innovation incentive and continuous improvement require establishing innovation reward funds, encouraging nursing staff and management personnel to propose innovative risk prevention and control measures, solving newly emerging problems through cross-departmental discussions, forming closed-loop management of “risk identification-learning-innovation-application-evaluation.” This mechanism embodies the dynamism and development of resilience governance, enhancing system adaptive capacity through continuous learning and innovation.

The *full-element resilience coupling mechanism* integrates governance elements from macro, meso, and micro levels, forming system resilience through synergistic coupling among elements. At the macro-level, policy support is linked with supervision, social multi-party participation is coordinated, subsidies are linked with quality supervision, forming a governance pattern of government leadership, social participation, and market operation. At the meso-level, internal management coordination within older adult care institutions, team collaboration and communication complementarity, risk early warning and quality management integration form internal organizational coordination mechanisms. At the micro-level, nursing and older adults element interaction, physical environment and emergency supplies adaptation, personalized plan customization form an person- centered care system for older adults. This mechanism embodies the systematicness of resilience governance, enhancing overall system resilience through synergistic coupling among elements.

The *whole-process dynamic response mechanism* is a dynamic response mechanism covering the whole process of pre-event, during-event, and post-event based on continuous reaction and coordination capacity. In the pre-event prevention stage, establish a risk early warning indicator system, conduct full-staff training and drills, and use real-time monitoring data to predict risks. In the during-event response stage, activate intelligent monitoring and information platforms, implement cross-departmental coordinated response, transmit risk information in real-time, and quickly allocate resources. In the post-event recovery stage, conduct facility and equipment repair, provide psychological counseling and support, and carry out review summary and improvement. This mechanism embodies the whole-process nature of resilience governance, forming a complete governance chain through organic connection of pre-event, during-event, and post-event stages.

These three mechanisms support and promote each other, jointly constituting the dynamic system of the resilience governance system. The adaptive proactive circulation mechanism provides system learning and innovation capacity; the full-element resilience coupling mechanism provides system coordination and integration capacity; the whole-process dynamic response mechanism provides system continuous response capacity. The coordinated operation of the three mechanisms enables the resilience governance system to effectively respond to complex and variable care risks.

## 5 Implementation pathways: subject coordination, policy support, and digital empowerment

The construction of a resilience governance system requires implementation through specific pathways. Based on the theoretical analysis above and drawing on international experience, this paper proposes a trinity implementation pathway: strengthening resilience governance subject coordination, improving resilience governance policy support, and digital technology empowerment of resilience governance.

**Figure 4:**
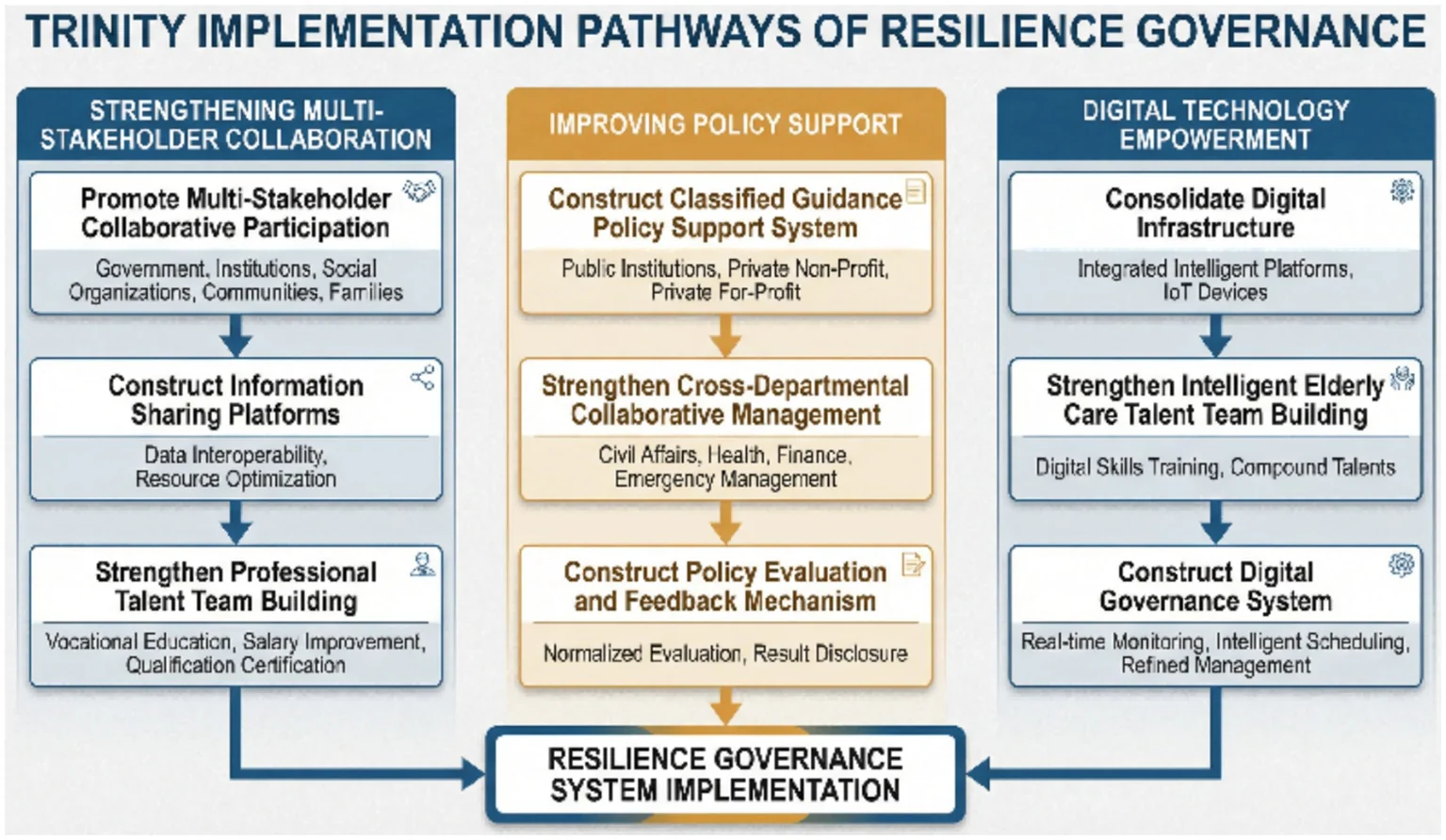
Trinity implementation pathways of resilience governance.

Figure 4 presents the trinity implementation pathways of the resilience governance system. The left pathway focuses on strengthening multi-subject coordination, including promoting multi-subject collaborative participation, constructing information sharing platforms, and strengthening professional talent team building; the middle pathway focuses on improving policy support and guarantee, including constructing classified guidance policy support systems, strengthening cross-departmental coordinated management, and constructing policy evaluation feedback mechanisms; the right pathway focuses on digital technology empowerment of governance, including consolidating digital infrastructure, strengthening smart older adult care talent team building, and constructing digital governance systems. The three pathways converge to form resilience governance system implementation.

### 5.1 Strengthening resilience governance subject coordination

The effectiveness of resilience governance depends on the collaborative participation of multiple subjects. In care risk governance in older adult care institutions, it is necessary to promote collaborative cooperation among multiple subjects including government, older adult care institutions, social organizations, communities, families, and older adults themselves.

First, *promote multi-subject collaborative participation*. Government plays a leading role in resilience governance and needs to provide policy support and resource guarantee. According to the “14th Five-Year Plan for Civil Affairs Development” issued by China’s Ministry of Civil Affairs, by 2025 (19), the total number of older adult care service beds will reach more than 9 million, and the proportion of nursing-type beds in older adult care institutions will reach more than 55%. To achieve this goal, government needs to increase fiscal investment, improve subsidy policies, and strengthen supervision and management. Social organizations can undertake professional services and social mobilization functions, such as providing older adult care service assessment, nursing staff training, and psychological support for older adults. Enterprises (older adult care institutions) need to inject capital and technology, improving service quality and management level. Communities can provide neighborhood mutual assistance and social support, creating a friendly older adult care environment. Families and older adults themselves are participants and beneficiaries of resilience governance and need to actively participate in care decision-making and risk prevention and control.

Second, *construct information sharing platforms*. Information asymmetry is an important obstacle constraining multi-subject coordination. Establishing unified and efficient information sharing mechanisms can achieve real-time data interconnection and business coordination, optimizing resource allocation. For example, establish comprehensive older adult care service information platforms, integrating older adults’ health records, older adult care institution service information, community resource information, and government policy information, providing decision support for all parties. Use big data technology to analyze health trends and risk status of older adults, providing basis for precise services and precise governance.

Third, *strengthen professional talent team building*. Nursing staff are direct providers of care services in older adult care institutions, and their professional capabilities directly affect care quality and risk prevention and control effectiveness. However, currently China’s older adult care nursing staff generally face problems such as insufficient numbers, low professional levels, and high mobility. According to the “Research Report on Older adults’’ Demand for Older adult Care Service Personnel from a Demand-Side Perspective” issued by the China Association of Social Welfare, China’s older adult care nursing staff shortage reaches 6 million (20). Strengthening professional talent team building requires multiple approaches: first, improve vocational education and training systems, enhancing nursing staff’s professional skills and service awareness; second, improve salary and career development channels, increasing occupational attractiveness; third, establish vocational qualification certification and continuing education systems, promoting professional development; fourth, strengthen vocational ethics education, cultivating nursing staff’s professional spirit and humanistic care capacity.

### 5.2 Improving resilience governance policy support

Policy support is an important support for the effective operation of the resilience governance system. It is necessary to improve policy support from policy support systems, cross-departmental coordinated management, and policy evaluation and feedback.

First, *construct classified guidance policy support systems*. China’s older adult care institutions include different types such as public institutions, private non-profit institutions, and private for-profit institutions. Different types of institutions have differences in operational models, funding sources, and service objects, requiring classified guidance policy support. For public institutions, fiscal investment should be strengthened to ensure they undertake basic older adult care services and bottom-line guarantee functions. For private non-profit institutions, support such as tax exemptions, construction subsidies, and operational subsidies should be provided, encouraging them to provide inclusive older adult care services. For private for-profit institutions, market mechanisms should guide them to provide diversified and personalized older adult care services, while strengthening supervision to ensure service quality and safety.

Second, *strengthen cross-departmental coordinated management*. Care risk governance in older adult care institutions involves multiple departments including civil affairs, health, finance, emergency management, and market supervision, requiring strengthening communication and collaboration among departments to ensure policy coherence and consistency. For example, civil affairs departments are responsible for admission approval and daily supervision of older adult care institutions; health departments are responsible for medical and health services and pandemic prevention and control; finance departments are responsible for funding support and subsidy distribution; emergency management departments are responsible for production safety and emergency response; market supervision departments are responsible for food safety and consumer rights protection. Establish cross-departmental coordination mechanisms, regularly convene joint meetings, coordinate to solve problems in policy implementation, forming governance synergy.

Third, *construct policy evaluation and feedback mechanisms*. Policy effectiveness needs to be tested through evaluation, and evaluation results need to be fed back into policy formulation and adjustment. Establish normalized evaluation systems, regularly evaluate the implementation status, effects, and problems of older adult care policies, forming closed-loop management of “formulation-implementation-evaluation-improvement.” Evaluation content should include multiple dimensions such as policy coverage, fund use efficiency, service quality improvement, and risk prevention and control effectiveness. Evaluation methods should combine quantitative analysis and qualitative analysis, focusing not only on statistical data but also on the actual feelings of older adults and nursing staff. Evaluation results should be promptly disclosed, subject to social supervision, and used as basis for policy adjustment.

Ethical and Privacy Considerations in Digital Empowerment: While digital systems offer significant benefits, they also introduce ethical and privacy risks. Implementation must incorporate privacy-by-design principles, including transparent data collection policies, regular privacy impact assessments, and resident/family involvement in system design. Digital systems should enhance rather than replace human care, prioritizing resident autonomy and dignity while ensuring compliance with data protection regulations.

### 5.3 Digital technology empowerment of resilience governance

Digital technology provides new tools and means for care risk resilience governance in older adult care institutions. By consolidating digital infrastructure, strengthening smart older adult care talent team building, and constructing digital governance systems, the accuracy, timeliness, and effectiveness of resilience governance can be enhanced.

First, *consolidate digital infrastructure*. Create integrated smart older adult care service platforms, promoting service provision and management operations toward informatization and intelligentization transformation. Smart older adult care service platforms should integrate multiple functional modules, including health monitoring modules, risk early warning modules, emergency response modules, service management modules, and data analysis modules. Health monitoring modules collect older adults’ vital sign data, activity data, sleep data, etc. in real-time through wearable devices and sensors, providing basis for health management and risk early warning. Risk early warning modules identify health risks and safety risks of older adults through big data analysis and artificial intelligence algorithms, promptly issuing early warning signals. Emergency response modules quickly activate emergency procedures when risks occur, allocating resources and coordinating forces from all parties. Service management modules support functions such as care plan formulation, service recording, and quality assessment. Data analysis modules conduct deep mining of accumulated data, discovering patterns and providing support for decision-making.

Second, *strengthen smart older adult care talent team building*. The application of digital technology requires professional talent support. Cultivating compound talents who understand both older adult care business and master digital skills is key to promoting smart older adult care development. On one hand, add digital technology courses to older adult care service professional education, cultivating students’ digital literacy and technology application capacity. On the other hand, conduct digital skills training for in-service nursing staff and management personnel, enhancing their capacity to use intelligent equipment, analyze data, and optimize processes. In addition, it is also necessary to introduce information technology professionals to provide technical support for the development, maintenance, and optimization of smart older adult care platforms.

Third, *construct digital governance systems*. Build digital governance platforms supporting whole-process management, achieving real-time monitoring, intelligent scheduling, and refined management. Digital governance platforms should have the following functions: first, real-time monitoring function, collecting operational data, environmental data, and older adults’ health data of older adult care institutions in real-time through IoT devices and sensor networks, forming a comprehensive data picture. Second, intelligent scheduling function, intelligently allocating nursing staff, medical resources, emergency supplies, etc. according to real-time data and prediction models, improving resource utilization efficiency. Third, refined management function, discovering weak links in management through data analysis, optimizing processes, and improving management level. Fourth, decision support function, providing data visualization tools and decision recommendations for managers, supporting scientific decision-making.

## 6 International experience and implications for China

### 6.1 International COVID-19 responses: lessons for resilience governance

#### 6.1.1 Global experience of older adult care institutions in responding to COVID-19

The COVID-19 pandemic caused enormous impact on global older adult care institutions. According to a systematic review study that analyzed 22 qualitative studies and 4 mixed-method studies covering 906 participants, the main challenges faced by older adult care institutions during the pandemic were identified as: difficulties in implementing pandemic prevention and control, resource shortages (personal protective equipment, medical supplies, professional personnel), negative emotions and physical burden of staff, insufficient departmental communication and coordination, and lack of support (5).

Facing these challenges, global older adult care institutions adopted various response strategies. Quantitative evidence highlights the effectiveness of specific resilience measures: (1) The joint implementation of rapid detection and isolation protocol scan significantly reduce the scale of an epidemic outbreak (21). (2) The cross-departmental collaboration strategy significantly enhanced the system’s resilience and played a positive role in reducing the virus transmission rate (22). (3) Digital monitoring systems: Facilities using AI-driven symptom tracking detected outbreaks 2–3 days earlier than traditional methods, enabling faster containment (23). (4) Policy effectiveness: Countries like New Zealand and Finland, which integrated older adult care institutions into national pandemic plans, achieved lower mortality rates (5) Lessons from failures: In contrast, lack of resilience measures led to catastrophic outcomes in countries like Belgium and Spain, where >50% of COVID-19 deaths occurred in care homes due to delayed lockdowns and inadequate staffing. These evidence-based outcomes demonstrate resilience governance in action.

Effective strategies include: first, role adaptation and redefinition, with staff undertaking additional tasks to ensure continuous care, flexibly adjusting work responsibilities, and conducting cross-professional collaboration. Second, innovative solutions and technology applications, adopting telemedicine services, applying digital tools, and reducing infection risks. Third, work organization and cooperation, strengthening team collaboration, improving work efficiency, and reducing staff workload. Fourth, positive psychological services, providing mental health support, implementing stress relief measures, and promoting staff health. Fifth, seeking community and organizational support, linking community resources, obtaining organizational-level support, and establishing effective communication mechanisms. Sixth, improving infection prevention awareness, conducting continuous training, updating knowledge, and strengthening prevention and control awareness (5).

Based on these experiences, researchers proposed future preparedness recommendations: first, enhance communication and decision-making capacity, establish rapid response mechanisms, optimize decision-making processes, and strengthen information sharing. Second, optimize material supply channels and physical space, establish emergency material reserves, optimize space layout, and improve infrastructure. Third, increase medical-related teams and training, expand professional personnel teams, strengthen emergency training, and enhance professional capacity (5).

#### 6.1.2 International best practices

Scandinavian countries (Norway, Sweden, Denmark, Finland) have internationally leading levels in older adult care institution design and service quality. older adult care institutions in these countries emphasize high-quality design standards, social sustainability, and inclusive environment creation. Specifically, they focus on age-friendly, barrier-free, and humanized design, emphasize social participation and quality of life of older adult care, and create supportive and resilient living environments (24).

Canada has launched a series of initiatives in resilience support for long-term care institutions. A report by Health Quality Ontario pointed out that Canada provides psychological trauma support for nursing assistants caring for older adults, conducts staff resilience training and support programs, and strengthens institutions’ capacity to respond to emergencies. These initiatives help enhance the psychological resilience and occupational resilience of nursing staff, thereby enhancing organizational resilience.

The U.S. Centers for Medicare & Medicaid Services (CMS) has formulated emergency preparedness plan standards for older adult care institutions. Research shows that emergency preparedness measures help increase the resilience of older adult care institutions, but gaps still exist among institutions in actual operations, with factors such as human resources, information maintenance, and communication collaboration affecting resilience (25). This experience suggests that formulating standards is only the first step; more importantly, ensuring effective implementation and continuous improvement of standards.

#### 6.1.3 Implications for resilience governance in China’s older adult care institutions

Drawing on international experience and combining with China’s national conditions, the construction of care risk resilience governance systems in China’s older adult care institutions can be advanced from the following aspects:

First, *draw on international experience and combine with China’s national conditions*. Learn from Scandinavian countries’ high-quality design standards, focusing on age-friendly design and humanized environment creation in older adult care institution construction. Reference U.S. emergency preparedness standard systems, formulating emergency preparedness norms for older adult care institutions that conform to China’s reality. Absorb Canada’s staff resilience support experience, strengthening psychological support and career development support for nursing staff. At the same time, full consideration must be given to China’s political system, economic development level, and social and cultural characteristics, giving play to government’s leading role, mobilizing multiple social forces, and constructing a resilience governance system with Chinese characteristics.

Second, *focus on multi-dimensional resilience construction*. In terms of spatial resilience, optimize the physical environment and infrastructure of older adult care institutions, ensuring architectural design meets safety standards and facilities and equipment meet emergency needs. In terms of institutional resilience, improve laws and regulations related to older adult care services, formulate detailed emergency plans, and establish sound supervision systems. In terms of organizational resilience, enhance organizational management capacity of older adult care institutions, strengthen team building, and optimize resource allocation. In terms of technological resilience, strengthen digitalization and intelligentization construction, using information technology to enhance risk monitoring, early warning, and response capacity. In terms of psychological resilience, focus on the mental health of nursing staff and older adults, provide psychological support services, and create a positive organizational culture.

Third, *construct a resilience governance system with Chinese characteristics*. Give play to government’s leading role, promoting resilience governance system construction through policy guidance, funding support, and supervision and management. Mobilize multiple social forces, encouraging social organizations, enterprises, communities, and families to participate in older adult care services and risk governance. Strengthen grassroots community participation, giving play to communities’ foundational role in older adult care services, establishing community-institution collaborative older adult care service networks. Promote digital technology empowerment, using big data, artificial intelligence, IoT, and other technologies to enhance governance capacity. Focus on cultural adaptability, fully considering Chinese cultural factors such as filial piety culture and family concepts in resilience governance system design, making the governance system more aligned with Chinese older adults’’ values and lifestyles.

Finally, *strengthen international exchange and cooperation*. Participate in the formulation of international older adult care service standards, share Chinese experience, and learn advanced international concepts. Conduct cross-national comparative research, deepening understanding of different countries’ older adult care institution resilience governance models. Establish international cooperation networks, promoting knowledge sharing and technology transfer. Through international exchange and cooperation, continuously enhance the level of care risk resilience governance in China’s older adult care institutions.

## 7 Limitations and future research

This paper presents a theoretical policy discussion and conceptual framework. The main limitation is the absence of empirical validation. Future research should focus on empirical testing of the proposed model through comparative case studies, development of validated assessment tools for the identified mechanisms, and longitudinal evaluation of framework implementation in diverse institutional settings.

## 8 Conclusion

This paper constructs a resilience governance system for care risks in older adult care institutions based on social-ecological systems theory, proposing a three-dimensional construction strategy of “concept-element-mechanism” and a trinity implementation pathway of “subject coordination-policy support-digital empowerment.” Research indicates that the resilience governance system from a social-ecological perspective can effectively respond to complex and variable care risks faced by older adult care institutions, providing theoretical basis and practical guidance for constructing a sustainable older adult care service system.

The main contributions of this paper are manifested in the following aspects:

First, *theoretical innovation*. This paper combines social-ecological systems theory with resilience theory, providing a new theoretical perspective for research on risk governance in older adult care institutions. Traditional risk governance research is mostly based on single-disciplinary perspectives, lacking systematicness and integration. Social-ecological systems theory emphasizes the multi-level structure, dynamism, complexity, and adaptability of systems, providing an integrative analytical framework for understanding the generation mechanism and evolution patterns of care risks in older adult care institutions. Resilience theory emphasizes system absorption capacity, adaptive capacity, and transformation capacity when facing shocks, providing theoretical guidance for constructing dynamic and adaptive governance systems. The combination of the two opens up new theoretical space for research on risk governance in older adult care institutions.

Second, *framework innovation*. This paper constructs a three-dimensional integrated resilience governance framework of “concept-element-mechanism.” At the concept level, three core concepts are proposed: development-security co-construction, temporal–spatial integration, and action-governance alignment, providing value orientation for the resilience governance system. At the element level, construction elements of resilience governance are systematically elaborated from macro, meso, and micro levels, forming a multi-level governance architecture. At the mechanism level, three operational mechanisms are proposed: adaptive proactive circulation mechanism, full-element resilience coupling mechanism, and whole-process dynamic response mechanism, providing dynamic support for the effective operation of the resilience governance system. This framework embodies logical levels from abstract to concrete, from concept to practice, with strong systematicness and operability.

Third, *pathway innovation*. This paper proposes a trinity implementation pathway of “subject coordination-policy support-digital empowerment.” Subject coordination emphasizes collaborative participation of multiple subjects, promoting collaborative governance through constructing information sharing platforms and strengthening professional talent team building. Policy support emphasizes classified guidance policy support systems, cross-departmental coordinated management, and policy evaluation and feedback mechanisms. Digital empowerment emphasizes enhancing governance capacity through consolidating digital infrastructure, strengthening smart older adult care talent team building, and constructing digital governance systems. This pathway embodies the organic combination of governance subjects, governance institutions, and governance technology, providing specific guidance for the implementation of the resilience governance system.

This study also has certain limitations. First, this paper is mainly based on literature research and theoretical analysis, lacking empirical data support. Future research can conduct empirical testing of the effectiveness of the resilience governance system through case studies, questionnaire surveys, and experimental research. Second, the resilience governance framework proposed in this paper is relatively macro and needs to be refined and adjusted according to the characteristics of different types of older adult care institutions in specific implementation. Future research can conduct classified research for different types such as public institutions, private institutions, and community-embedded institutions. Third, this paper mainly focuses on resilience governance within older adult care institutions, with insufficient attention to the interactive relationship between older adult care institutions and external environments (such as communities, medical institutions, and government departments). Future research can explore resilience construction of older adult care institutions in broader older adult care service networks from a network governance perspective.

Despite these limitations, the research in this paper provides a systematic theoretical framework and practical guidance for care risk resilience governance in older adult care institutions, having important significance for promoting the modernization of China’s older adult care service system. As population ageing deepens, care risk governance in older adult care institutions will face more challenges and opportunities. We look forward to more scholars and practitioners paying attention to this field, jointly promoting the in-depth development of research and practice on resilience governance in older adult care institutions, providing safer, higher-quality, and more sustainable care services for older adults.
